# Synthetic Microbial Community Biosensors: From Engineered Ecosystems to Modular Detection Platforms with AI-Driven Intelligence

**DOI:** 10.3390/bios16070366

**Published:** 2026-07-06

**Authors:** Liangshu Hu, Yipei Yang, Shiqi Xia, Wenhui Mao, Ying Shang, Yuzhen Wang, Huijuan Yang, Mingzhang Guo

**Affiliations:** 1 Key Laboratory of Digital-Intelligence and Dynamic Perception for Food Quality of China Light Industry, Beijing Technology and Business University, Beijing 100048, Chinaguomingzhang@btbu.edu.cn (M.G.); 2 Beijing Laboratory for System Engineering of Carbon Neutrality, Beijing Municipal Education Commission, Beijing 100048, China; 3 Faculty of Food Science and Engineering, Kunming University of Science and Technology, Kunming 650500, China

**Keywords:** whole-cell biosensors, microbial consortia, synthetic microbial community biosensors, artificial intelligence

## Abstract

Synthetic microbial community (SynCom) biosensors are emerging from the convergence of whole-cell biosensing, synthetic ecology, and computational design. Conventional whole-cell biosensors (WCBs) use a single microbial chassis to convert analyte recognition into optical, electrochemical, gaseous, or growth-linked outputs. This compact architecture supports low-cost and field-oriented detection, but it can be limited by cellular burden, narrow dynamic range, environmental interference, and difficulty in interpreting multicomponent signals. Natural microbial consortia provide an ecological template in which sensing, transformation, stress tolerance, and response are distributed across interacting populations. SynCom biosensors seek to translate this logic into engineered platforms with defined members, assigned functional roles, designed communication, and interpretable readouts. This review traces the transition from WCBs to natural consortia and engineered multicellular biosensors, emphasizing functional partitioning, signal routing, community control, and artificial intelligence (AI)-assisted design. AI is discussed as a practical tool for narrowing design space, predicting interactions, decoding complex biosignals, and supporting adaptive operation. Key challenges remain in community stability, orthogonal communication, data quality, biosafety, standardization, and real-sample validation. Future progress will depend on parsimonious community design, reliable containment, quantitative validation, and computational workflows that connect community composition with sensing performance.

## 1. Introduction

Environmental and food-safety monitoring increasingly requires detection systems that can operate in chemically complex and spatially heterogeneous matrices. Heavy metals, persistent organic pollutants, pesticide residues, antibiotics, and pathogenic microorganisms rarely occur as isolated hazards. They often coexist in soils, sediments, wastewater, agricultural products, and biological samples, where matrix effects and mixture toxicity can obscure the relationship between total contaminant concentration and biological risk [1,2,3]. Instrumental methods such as atomic absorption spectroscopy, inductively coupled plasma mass spectrometry, gas chromatography-mass spectrometry, liquid chromatography-mass spectrometry, and polymerase chain reaction remain central to confirmatory analysis because they provide high analytical sensitivity, chemical resolution, and standardized quantification. Their limitations are mainly operational. Most require centralized laboratories, costly equipment, trained personnel, sample transport, and pretreatment steps that reduce temporal resolution and restrict field deployment [4,5]. These constraints are consequential during industrial leakage, pathogen contamination, pesticide misuse, and contamination events across food supply chains.

Whole-cell biosensors (WCBs) provide a complementary strategy by using living cells to convert analyte recognition into measurable outputs such as fluorescence, bioluminescence, electrochemical signals, gas reporters, or growth-linked phenotypes [6]. Because cells respond to the bioavailable fraction of a compound and contain endogenous signal amplification mechanisms, WCBs can provide functional information that chemical measurement alone may not capture. Early genetically engineered Escherichia coli reporters for antimonite and arsenite demonstrated the feasibility of coupling metal-responsive regulatory systems to analytical outputs [7]. Subsequent WCBs have incorporated synthetic promoters, engineered transcription factors, riboswitches, logic circuits, memory devices, and containment modules [8,9]. These advances have expanded sensing capacity, but they have also exposed the limits of the single-chassis format. One cell must carry analyte uptake, recognition, signal processing, reporter expression, growth maintenance, and stress response. Additional targets or logic functions often increase circuit burden, crosstalk, evolutionary instability, and false-positive behavior, especially in matrices where pH, temperature, competing ions, organic matter, and endogenous metabolites perturb sensing circuits [10,11].

Natural microbial consortia suggest a different architecture. In soil, biofilms, gut communities, and contaminated environments, microorganisms distribute functions across interacting populations through metabolic division of labor, cross-feeding, quorum sensing, syntrophy, and stress buffering [12,13]. Such communities can process complex substrates and maintain activity under fluctuating conditions more effectively than many isolated strains. However, natural consortia are not automatically suitable as analytical devices. Their membership, interactions, and outputs are often context-dependent, difficult to standardize, and poorly aligned with quantitative detection. Synthetic microbial community (SynCom) biosensors address this gap by translating ecological principles into engineered systems. In this context, a SynCom is not simply a natural consortium with fewer members, but a deliberately assembled community with defined composition and intended functions. A SynCom biosensor further narrows this concept to detection, because its members are assigned roles in recognition, transformation, communication, reporting, or containment so that an analyte or environmental cue can be converted into an interpretable output. In this architecture, community composition becomes a design variable rather than a background condition [14]. Ecological processes such as division of labor, cross-feeding, quorum sensing, spatial organization, and robustness therefore become relevant to biosensing when they can be translated into controllable design features, including functional assignment, signal routing, community control, and analytical readout. The central design question changes from how much circuitry can be placed in one chassis to how a sensing task should be distributed across a stable and interpretable multicellular system. Artificial intelligence (AI) and machine learning add a further layer to this progression. They can support candidate strain selection, interaction prediction, model-guided circuit design, signal decoding, and closed-loop community control [15]. Their role should not be overstated: current datasets are uneven, interaction mechanisms remain incompletely understood, and model transferability across environments is limited. Nevertheless, AI-assisted workflows may help convert SynCom biosensors from empirical assemblies into more predictable detection platforms.

This review examines the transition from single-cell biosensors to AI-assisted SynCom biosensors (Table 1). Here, WCBs are not treated as an independent endpoint, but as the technical and conceptual baseline from which distributed biosensing architectures can be understood. The review first identifies the single-chassis constraints that limit conventional WCBs, then analyzes natural microbial consortia as ecological templates for distributed sensing. It subsequently discusses design principles and control strategies for SynCom biosensors and evaluates how AI can assist design, decoding, and adaptive operation. The overall aim is to clarify where SynCom biosensors offer genuine conceptual advantages and where substantial engineering and translational barriers remain.

## 2. From WCBs to Distributed Biosensing: Rationale for SynCom Biosensor Architectures

### 2.1. Single-Cell Biosensing as the Baseline Architecture

WCBs provide the historical and technical foundation for SynCom biosensors. A WCB is a living analytical system in which a microbial cell recognizes an environmental or physiological input and converts that recognition event into a measurable output. Compared with biosensors based on purified enzymes, antibodies, or nucleic acids, WCBs integrate analyte recognition, signal conversion, biological amplification, and cellular context within a self-replicating unit [6,36]. This feature makes them attractive for environmental monitoring, food safety, bioprocess surveillance, and screening of bioavailable contaminants.

For the purpose of SynCom biosensor design, WCBs can be viewed as a compact architecture in which three functions are compressed into a single chassis. The recognition function detects the target through transcription factors, riboswitches, two-component systems, enzymes, transporters, or other microbial recognition elements [16,37,38,39,40,41,42]. The intracellular processing function converts recognition into gene expression, metabolic change, recombinase activity, memory recording, or another regulatory event [43,44,45]. The output function transforms the biological response into an analytical readout, including fluorescence, bioluminescence, electrochemical signals, gaseous reporters, magnetic enrichment, or growth-linked phenotypes [46,47,48,49,50]. This modular view is important because it identifies which operations are normally integrated within one cell and which operations could, in principle, be redistributed across different community members.

The development of synthetic promoters, engineered transcription factors, riboswitches, logic circuits, memory devices, reporter proteins, and containment modules has greatly expanded the capability of WCBs [8,9,10,11]. These advances demonstrate that a single microbial chassis can be engineered into a versatile sensing device. At the same time, they reveal a central design tension: as more recognition, processing, reporting, and safety functions are loaded into one cell, the chassis increasingly becomes both the analytical platform and the limiting resource. This tension provides the conceptual starting point for SynCom biosensors, in which sensing functions are no longer assumed to reside entirely within one organism.

### 2.2. Single-Chassis Constraints That Motivate Distributed Sensing

The main constraints of WCBs arise from the same feature that makes them powerful: all analytical functions are embedded in one living cell. Sensitivity and specificity are often difficult to optimize simultaneously. Stronger promoters, higher regulator expression, or signal amplification may increase output intensity, but they can also elevate background activity and false-positive responses. Conversely, stricter repression or reduced leakiness may improve specificity at the cost of signal magnitude, response speed, or usable dynamic range [10,43].

Physiological burden further restricts the scalability of single-cell biosensing. A single chassis must maintain growth, analyte uptake, recognition, signal processing, reporter expression, stress tolerance, and genetic stability at the same time. When additional targets, logic operations, or containment modules are introduced, intracellular resource competition becomes more pronounced. Regulatory interference, delayed signal propagation, metabolic cost, and evolutionary pressure for circuit silencing or plasmid loss can all compromise long-term performance. These issues are particularly relevant for field-oriented biosensors, where matrix stress and operational stability may be as important as peak signal intensity.

Environmental complexity compounds these single-chassis constraints. Real samples such as soils, wastewater, food matrices, sediments, and biological materials contain competing ions, organic matter, endogenous metabolites, variable pH, fluctuating temperature, turbidity, and background microbial populations. These factors can alter cell physiology, analyte bioavailability, promoter activity, reporter maturation, and signal acquisition. Under such conditions, a change in biosensor output may reflect the target analyte, but it may also reflect host stress, matrix inhibition, growth-state variation, or nonspecific pathway activation [10,11].

Multiplexed detection exposes the same problem in a more pronounced form. Single-cell platforms can be engineered to process multiple inputs, but spectral overlap, regulatory crosstalk, resource competition, and unstable expression become increasingly difficult to control as more recognition modules, logic gates, and reporters are assembled in the same chassis. These bottlenecks suggest that some biosensing tasks may not be best solved by further increasing the complexity of one cell. Instead, they may require a distributed architecture in which recognition, transformation, computation, reporting, and stabilization are assigned to several defined populations.

### 2.3. Design Implications for SynCom Biosensors

The transition from WCBs to SynCom biosensors should therefore be understood as an architectural shift rather than a simple increase in biological complexity. In a single-cell biosensor, the central design question is how many functions can be engineered into one chassis without compromising sensitivity, specificity, or stability. In a SynCom biosensor, the question changes to how a detection task should be partitioned across interacting members so that each population performs a defined and controllable role.

This shift creates several design opportunities. A transformation strain can convert a poorly sensed analyte into a detectable intermediate. A tolerant strain can buffer toxicity or remove inhibitory byproducts. A reporter strain can be optimized for signal generation rather than degradation or stress resistance. Communication modules can route information between populations, while containment or stabilizing modules can improve persistence and reduce uncontrolled spread. In this sense, SynCom biosensors do not replace WCBs; they reorganize the functional logic of WCBs at the community level.

This rationale also clarifies the transition to natural microbial consortia. Natural communities already distribute substrate recognition, metabolic transformation, communication, stress buffering, and collective response across interacting populations. They are not standardized analytical devices, but they reveal ecological principles that can be translated into engineered SynCom biosensors. The next section therefore examines natural microbial consortia as conceptual templates for distributed biosensing, with particular attention to division of labor, cross-feeding, community communication, spatial organization, and robustness.

## 3. Natural Microbial Consortia as Ecological Foundations for Distributed Biosensing

Natural microbial consortia are not biosensors in the strict synthetic-biology sense because they do not usually transform environmental inputs into deliberately standardized and instrument-readable outputs. Yet many natural communities already perform operations that resemble distributed sensing. Environmental recognition, substrate conversion, signal exchange, stress buffering, and collective response are often partitioned across interacting partners rather than confined to one cell. This ecological arrangement allows communities to process chemically complex, spatially heterogeneous, and dynamically fluctuating inputs more effectively than a single microbial chassis. Natural consortia therefore provide a conceptual bridge between classical WCBs and engineered synthetic communities. Their value lies not in serving as ready-made analytical devices, but in revealing ecological operations that can be abstracted into designed biosensing architectures [12,13,17].

### 3.1. Division of Labor and Metabolic Relay

**Figure 1:**
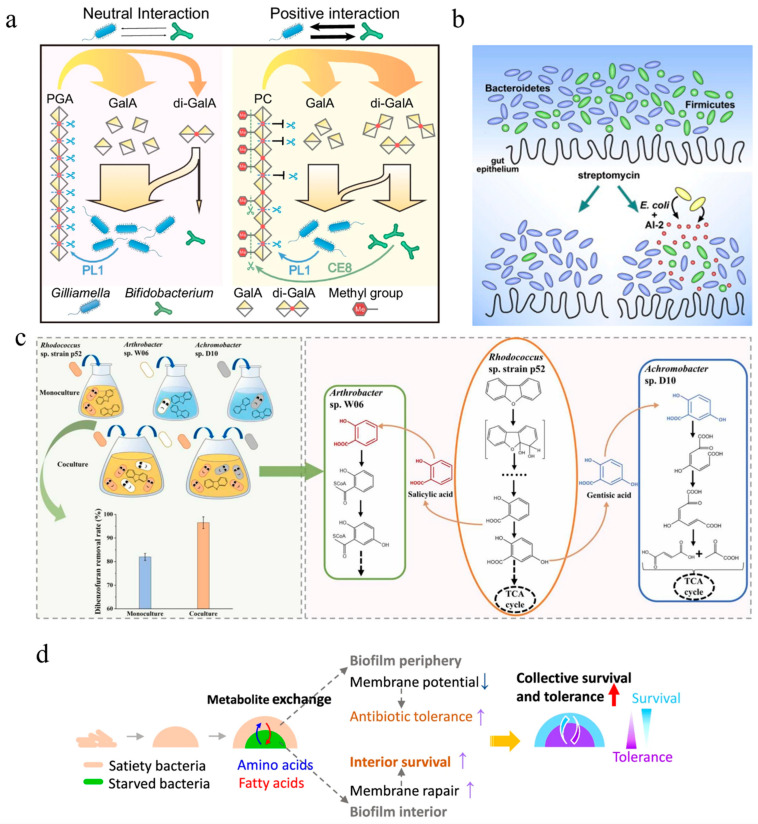
Natural microbial consortia as ecological templates for distributed biosensing. (**a**) Dietary-dependent interaction between *Gilliamella* and *Bifidobacterium* illustrates how substrate preprocessing by one member can support the function of another member [18]. (**b**) AI-2-mediated remodeling of the antibiotic-treated gut microbiota shows that intercellular signals can reshape community state, rather than simply report a single target [52]. (**c**) Dibenzofuran degradation by *Rhodococcus* sp. strain p52 and partner strains illustrates metabolic relay through intermediate exchange and supporting catabolism [19]. (**d**) Spatial metabolite exchange (amino acids and fatty acids) between biofilm interior and periphery shows how structured communities generate local niches and exchange zones [21].

A central mechanism behind this advantage is division of labor. For biosensor design, division of labor is valuable because it separates pathway burden, substrate accessibility, toxicity management, and signal attribution into different cellular contexts. In a single cell, target recognition, uptake, metabolism, detoxification, and signal reporting compete for the same intracellular resources. Natural alliances instead distribute these tasks among specialists. This principle is evident in the honeybee gut microbiota, where *Bifidobacterium* and *Gilliamella* act as major degraders of plant polysaccharides and partition pectin and hemicellulose digestion across phylogenetically distinct but functionally complementary partners [51]. Mechanistic work further showed that mutualistic interaction between *Bifidobacterium asteroides* and *Gilliamella apicola* (Figure 1a) depends on synergistic pectin deconstruction. When the two strains co-occur, *Bifidobacterium* provides complementary demethylation that promotes *Gilliamella* growth on methylated homogalacturonan [18]. For SynCom biosensors, the lesson is direct. A target does not always need to be recognized and fully processed by one cell. One population may convert a chemically inaccessible input into a tractable intermediate, while another population performs recognition or reporting. Such partitioning can expand the detectable chemical space beyond the physiological limits of a monoculture, provided that the relay remains traceable.

Cross-feeding then converts specialization into integrated community function. In many natural consortia, one population does not simply consume its preferred substrate, but releases metabolites that become growth substrates, detoxification sinks, or informational intermediates for neighboring members. Such metabolic relays are highly relevant to biosensor thinking because they offer a route for distributed analyte preprocessing. A recent study on dibenzofuran degradation illustrates this point well (Figure 1c). During synergistic catabolism, *Rhodococcus* sp. strain p52 released intermediates such as salicylic acid and gentisic acid, which were then utilized by *Arthrobacter* sp. W06 and *Achromobacter* sp. D10, respectively. Pairwise coculture enhanced both community growth and pollutant degradation, while coordinated transcriptional regulation across partners prevented inhibitory accumulation of toxic intermediates [19]. In a biosensing framework, this means that one member can broaden target accessibility, a second can remove toxic intermediates that would otherwise suppress system performance, and the consortium as a whole can achieve a more stable and informative response than a solitary cell. Such distributed metabolic processing is particularly attractive for complex pollutants, mixed substrates, and chemically recalcitrant analytes that overwhelm conventional WCB architectures. The analytical implication is not simply that more species improve degradation. Rather, a useful metabolic relay must preserve a traceable relationship between upstream conversion and downstream response. If intermediates accumulate, diffuse away, or are consumed by nonreporting members, distributed sensing may weaken analytical accuracy even while expanding target accessibility.

### 3.2. Community Communication and Spatial Organization

Natural alliances are also coordinated by community-level communication. Quorum sensing and related signaling processes enable populations to couple local metabolic events to collective behavioral outputs. In the gut, manipulation of the interspecies signal autoinducer-2 (AI-2) altered the composition of the antibiotic-treated mouse microbiota, favoring expansion of Firmicutes and shifting the *Firmicutes/Bacteroidetes* balance (Figure 1b), thereby demonstrating that signal exchange can reshape community structure under dysbiosis [52]. Other systems show the same principle in different contexts. In the oral cavity, *Aggregatibacter actinomycetemcomitans* activated the quorum-sensing regulon of Streptococcus mutans in dual-species biofilms, with strong induction of sigX and associated competence and mutacin genes occurring only in co-culture, not in isolated monocultures [53]. In wastewater-derived nitrifying communities, quorum sensing has been linked to granular sludge assembly, and functional metagenomics identified active luxI/luxR homologs in *Nitrospira*, indicating that key nitrifiers are embedded in community signaling rather than acting merely as passive metabolic players [54,55]. These examples indicate that the ‘sensor’ in a natural consortium is often not a single receptor-bearing cell, but a changing community state generated by interactions among members. In engineered biosensors, this creates a key distinction. Communication can coordinate community behavior, but it should not be equated with analyte-specific readout. Natural quorum signals often reflect community state rather than target concentration, and this ambiguity must be controlled in analytical design.

Spatial organization strengthens this distributed sensing logic. Biofilms and other structured microbial aggregates do not simply place cells next to one another; they create physicochemical gradients, localized niches, and metabolite exchange zones that stabilize otherwise incompatible metabolisms. Early work on microbial biofilms emphasized that spatial organization optimizes resource use and facilitates syntrophic processes or special microenvironments that are difficult to sustain in well-mixed populations [20]. Later analyses of physiological heterogeneity in biofilms further showed that nutrient and oxygen gradients create microscale stratification (Figure 1d), allowing distinct subpopulations to occupy different metabolic states within the same community [21]. In granular systems, this principle becomes even more evident, because spatial structure helps segregate slower and more sensitive populations from outer-layer competitors while preserving metabolite exchange across the consortium. For biosensors, spatial organization means that recognition, transformation, and response can be physically distributed rather than forced into a single intracellular space [56,57,58]. This can improve tolerance to environmental fluctuation and reduce competition between incompatible functions. At the same time, spatial structure can make performance dependent on diffusion distance, local density, and microenvironmental gradients. It is therefore a useful design variable, but also a source of analytical variability.

### 3.3. From Ecological Buffering to Analytical Robustness

Together, division of labor, communication, and spatial organization can provide ecological buffering that is difficult to achieve in conventional single-cell biosensors. However, ecological buffering should not be equated with analytical robustness. Host-associated microbiomes provide useful examples. On human skin, interspecies quorum sensing was shown to protect against epidermal injury in atopic dermatitis, indicating that community signaling can stabilize host-microbe interactions [59]. In another case, probiotic Bacillus abolished colonization by Staphylococcus aureus through signaling interference, showing that community members can modulate pathogen behavior by disrupting the communication logic required for colonization and virulence [60]. These examples suggest that microbial interactions may reduce sensitivity to environmental disturbance, competitor invasion, or local stress. They do not, however, prove that a community will generate a more reliable analytical signal.

For SynCom biosensors, robustness should be defined operationally as preservation of an interpretable input–output relationship under perturbation. Useful descriptors include signal retention under matrix stress, coefficient of variation across biological replicates, signal-to-background ratio, dynamic-range preservation, population-ratio drift, response and recovery time, and accuracy in blank, spiked, and mixed-target matrices. Resilience, by contrast, describes the recovery of community composition or sensing function after perturbation. Natural consortia often achieve ecological persistence through redundancy and feedback, but the same redundancy can weaken signal attribution. Ecological robustness therefore becomes useful for SynCom biosensing only when it is translated into measurable analytical robustness.

### 3.4. Conditional Interactions and Limits of Natural Consortia as Biosensing Templates

The same features that make natural microbial alliances powerful also define their limits as biosensor platforms. Division of labor expands chemical reach but creates transfer and attribution problems. Communication coordinates population behavior but may obscure analyte-specific readout. Spatial organization stabilizes exchange but makes performance geometry-dependent. Robustness buffers stress but may mask the signal component that should be measured. These limitations arise because natural interactions are conditional rather than programmable. In the honeybee gut, mutualism between *Bifidobacterium* and *Gilliamella* depends on the biochemical nature of the pectin substrate [18]. AI-2 reshapes gut community composition in a state-dependent manner under antibiotic perturbation rather than producing a fixed output [52]. In oral biofilms, quorum-sensing activation emerges only in the presence of a partner [53]. In granular sludge, community-level quorum sensing is entangled with quorum quenching, species succession, and biofilm assembly dynamics [54]. Thus, natural alliances solve the single-cell bottleneck through ecological complexity, but the same complexity reduces modularity, predictability, and controllability. Population ratios drift, communication channels crosstalk, spatial arrangements reorganize, and outputs are often diffuse physiological state changes rather than standardized reporter signals.

For SynCom biosensors, the relevant question is whether a natural interaction reveals a function that can be abstracted into a defined sensing architecture. Useful interactions should be linked to analyte conversion, signal transfer, stress buffering, community stabilization, or output formation. Interactions outside these categories are better treated as sources of uncertainty. Viewed schematically, the ecological template contributes four transferable operations, including functional partitioning, interaction-mediated signal flow, spatial buffering, and controlled conversion of ecological robustness into analytical robustness. SynCom biosensors emerge by retaining these operations while imposing defined membership, engineered interaction, and measurable output.

## 4. SynCom Biosensors: Precision Engineering for Next-Generation Biosensing

### 4.1. From Co-Cultures to Defined Biosensing Ecologies

SynCom biosensors are best understood as defined biosensing ecologies rather than as simple mixtures of reporter strains. Their members are selected or engineered to capture, transform, compute, and convert biological information into readable outputs, while the relationships among those members are part of the device architecture [14]. The analytical task is therefore not to reproduce natural complexity, but to compress selected ecological operations into minimal, controllable, and readable communities. A consortium becomes a SynCom biosensor only when community composition, functional assignment, interaction design, and signal acquisition are specified together. The construction workflow can be condensed into three linked tasks, including candidate selection, SynCom construction and validation, and biosignal detection through an appropriate interface. This workflow clarifies the position of SynCom biosensors between WCBs and engineered ecosystems. They retain genetically encoded specificity, distribute incompatible or burdensome functions across populations, and remain more compositionally defined than natural consortia.

**Figure 2:**
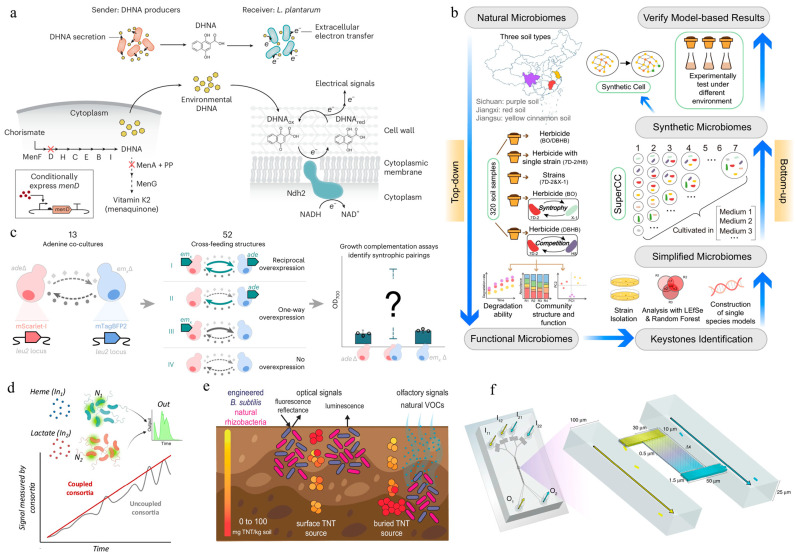
Engineering strategies that convert distributed microbial functions into SynCom biosensor architectures. (**a**) Mechanism of the e-COSENS system for modular bioelectronic sensing. The diagram illustrates the plug-and-play architecture consisting of a ‘sender’ bacterium that synthesizes the mediator DHNA in response to analytes and a ‘receiver’ bacterium (*L. plantarum*) that converts DHNA into electrical signals via the Ndh2-dependent extracellular electron transfer (EET) pathway [23]. (**b**) Function-enhanced synthetic microbiomes show how natural enrichment and bottom-up reconstruction can link ecological function with defined community design [63]. (**c**) A systematic screening framework for adenine-mediated cross-feeding, evaluating 52 unique consortia architectures by pairing an adenine auxotroph with 13 different metabolic auxotrophs across four interaction modes (reciprocal, one-way, and basal secretion) [68]. (**d**) A comparative framework of biosensor consortia demonstrating that coupled systems (red) significantly enhance output reliability and robustness against population fluctuations compared to uncoupled systems (gray) [28]. (**e**) Integrated framework and synthetic genetic circuitry of an autonomous microbial sensor for long-term TNT detection in natural soil [75]. (**f**) Schematic of the MISTiC microfluidic platform for investigating spatiotemporal dynamics in microbial consortia [77].

Two recent studies illustrate the transition from distributed detection as a concept to a practical biosensor architecture. Khatun et al. [22] constructed a bacterial consortium-based sensing system for organophosphorus pesticides in which one *Escherichia coli* strain hydrolyzed the pesticide into *p*-nitrophenol and a second strain converted this intermediate into a colorimetric β-galactosidase output. The analytical principle was not stronger reporter expression alone, but division of the assay into transformation and recognition modules. Li et al. [23] extended this logic into a modular bioelectronic co-culture architecture. In their e-COSENS system (Figure 2a), a sender bacterium produced electron mediators in response to analytes, whereas a receiver bacterium converted these mediators into electrical signals through extracellular electron transfer. By swapping sender strains and their sensing elements, the platform detected metals, small molecules and peptides across environmental, food, human-relevant and microbial-community samples. These examples show that SynCom biosensors can compute across populations when interaction design is treated as part of the sensing circuit rather than as background ecology.

### 4.2. Construction Logic: Top-Down, Bottom-Up, and Middle-Out Design

The first design decision is how the community will be assembled. Top-down construction begins with a complex natural community and uses enrichment, selective pressure, serial passage, or domestication to simplify it toward a desired phenotype. This strategy can preserve evolved ecological dependencies and may yield robust consortia for pollutant degradation or stress tolerance, but the causal mechanisms often remain opaque. Bottom-up construction begins with isolated strains whose traits are known, then assembles them into a defined community. This approach offers greater reproducibility and mechanistic clarity, but it can lose stabilizing interactions that would have been present in a natural assemblage [61,62].

For biosensor development, neither top-down nor bottom-up construction is sufficient by itself. A purely top-down biosensor risks weak analytical attribution because the measured output may reflect uncontrolled physiological changes. A purely bottom-up biosensor may be elegant but fragile if it ignores the ecological constraints that govern survival, growth, and signal exchange in the target matrix. A middle-out strategy is therefore more appropriate. In this strategy, natural communities or environmental enrichments identify candidate functions, keystone members, and interaction motifs, whereas bottom-up engineering defines the minimal membership, regulatory links, and measurable outputs required for a biosensor. This logic is increasingly visible in microbiome engineering. In herbicide-contaminated soils, Ruan et al. [63] combined functional microbiome enrichment, keystone-species identification and SuperCC-guided metabolic modelling to construct simplified synthetic microbiomes with enhanced bioremediation performance (Figure 2b). Similarly, Wu et al. [64] used TBBPA-degrading enrichments to identify keystone taxa and interaction patterns, then rebuilt a four-member synthetic consortium that retained degradation activity in soil. Although these systems were developed for bioremediation rather than sensing, they provide a useful design precedent for SynCom biosensors: ecological enrichment supplies the candidate biological functions, while engineering reconstruction converts them into defined, interpretable and controllable measurement systems.

The design principles summarized by Zhang et al. [62] provide a useful framework for this middle-out logic. SynCom construction should emphasize functional parsimony and modularization, prediction and control of interaction networks, robust and stable community behavior, and reduction in unnecessary complexity. These principles are directly transferable to biosensors. The best SynCom biosensor is not the largest community, but the smallest community that performs a detection task that a single chassis cannot complete reliably. Matuszyńska et al. [65] make a related point from a modeling perspective. They argue that microbial community design should shift from organism-centered thinking to modules of community function. For SynCom biosensors, this means that a strain is selected not because it is taxonomically interesting, but because it contributes a defined function, such as analyte uptake, intermediate generation, orthogonal communication, reporter output, matrix tolerance, or population stabilization. This functional view prevents SynComs from being treated as a fashionable extension of WCBs and instead frames them as a necessary architecture for specific analytical problems.

### 4.3. Functional Partitioning and Metabolic Relay

Functional partitioning is the most direct reason to build a SynCom biosensor. Recognition, transformation, amplification, computation, reporting, containment, and survival can be separated into strains with different physiological capacities. This reduces intracellular burden, permits incompatible pathways to operate in different cellular contexts, and allows the designer to use specialized organisms or engineered strains for distinct subtasks [66,67]. Synthetic consortia developed for bioproduction, although not biosensors in the strict sense, provide useful engineering case studies because they show how distributed metabolism can be made stable and programmable. Peng et al. [68] developed a molecular toolkit of cross-feeding yeast strains, demonstrating how defined metabolic dependencies can be used to assemble synthetic communities with programmed interdependence (Figure 2c). Rong et al. [69] used CRISPR interference to impose a synthetic anaerobic physiology under oxic conditions and coupled this strain to a second *E. coli* population that consumed the acetate byproduct, enabling two concurrent fermentations in one bioreactor. These studies extend the biosensor discussion by showing how metabolic interdependence, growth-state switching, and byproduct consumption can be used to stabilize functional partitioning in engineered communities.

For detection, metabolic relay has three implications. It can broaden the detectable chemical space by converting poorly sensed analytes into tractable intermediates, as shown by growth-coupled *Pseudomonas putida* sensor platforms that use engineered metabolic dependencies to detect PCA, D-lactate and PET-degradation products released by partner strains or enzymatic reactions [24]. It can insulate sensing from toxicity by assigning transformation to a tolerant or catalytically specialized strain and reporting to a more responsive strain, a logic illustrated by ethylene sensing systems in which monooxygenase-expressing cells convert ethylene into ethylene oxide before the reporter strain generates the fluorescent output [25]. It can also improve specificity when detection depends on a sequence of events rather than on a single promiscuous receptor. These advantages are valuable only if the relay is experimentally validated [70]. A SynCom biosensor should therefore report not only its limit of detection, but also the persistence of each member, the efficiency of intermediate transfer, the relay response time, and system behavior in mixed-target and real-sample conditions. This creates a concise evaluation scheme for relay-based SynCom biosensors, linking member stability, transfer fidelity, output specificity, and matrix robustness.

### 4.4. Communication, Crosstalk, and Signal Routing

A distributed biosensor requires communication, but communication in SynCom biosensors should be distinguished from computational coordination. In some systems, communication is metabolic: one member releases, transforms, or removes a molecule that another member consumes or senses. This mode supports relay-based detection when an analyte is first converted into a more detectable intermediate or when toxicity and reporting are assigned to different populations. In other systems, communication is regulatory: quorum-sensing signals, peptides, volatile molecules, or electrochemical outputs coordinate gene expression across populations. Regulatory communication controls activation thresholds, timing, or population-level behavior rather than simply transferring an analyte-derived intermediate. Computational coordination is a higher-level concept. It refers to the way metabolic and regulatory links are arranged so that the consortium produces a defined input–output behavior, rather than a simple sum of independent strain responses. Synthetic biology has provided several tools for these purposes, but the same communication systems that enable coordination can also generate crosstalk and instability [71,72].

The distinction between useful crosstalk and harmful crosstalk should be made explicit. Natural crosstalk can stabilize ecosystems or coordinate collective behavior, whereas analytical biosensors often require signal orthogonality so that one input does not activate another output channel. Kylilis et al. [26] addressed this problem by characterizing AHL receiver devices and identifying communication channels with limited crosstalk. Du et al. [27] extended this principle by constructing a de novo intercellular signaling toolbox with multiple orthogonal channels, enabling bacterial consortia to process several communication signals simultaneously and to implement distributed biocomputation across seven engineered strains. For SynCom biosensors, crosstalk should not be treated only as a defect. It can be used deliberately for global coordination, but it must not obscure analyte-specific readout. The heme-lactate consortia biosensor reported by Huang et al. [28] is useful because it shows how a shared regulatory signal can be engineered into the computational structure of the biosensor (Figure 2d). Instead of allowing each strain to respond independently, the system coupled biosensor activity to a global quorum-sensing signal and used an incoherent feedforward-loop design to improve robustness against population perturbations. This work addresses a central SynCom challenge, namely how to coordinate several responsive populations without allowing population imbalance to dominate the analytical signal.

Signal routing is the final step that makes distributed biology analytically useful. A SynCom biosensor should generate outputs that are attributable, nonoverlapping, and convertible into a quantitative or categorical result. This requires a clear connection between the type of communication used and the final readout: metabolic communication should preserve the relationship between analyte conversion and reporter activation, regulatory communication should coordinate expression without introducing nonspecific activation, and computational coordination should make the collective output more interpretable rather than more ambiguous. Optical reporters, gas reporters, electrochemical currents, growth signatures, metabolite ratios, and spatial patterns can all be used, but the output must be matched to the sample matrix. For opaque food, soil, or sludge samples, optical outputs may require extraction, imaging, or device-assisted separation, whereas electrochemical, gaseous, or magnetic outputs may reduce matrix interference. In all cases, reporter selection should be justified by the deployment environment rather than by convenience in laboratory assays.

### 4.5. Composition, Temporal Coordination, and Spatial Format as Control Variables

Control is the central engineering challenge once biosensing is distributed across multiple members. A single-strain biosensor can often be tuned through promoter strength, copy number, receptor affinity, or reporter maturation. A SynCom biosensor must also maintain the composition of the community, the timing of intercellular events, and the spatial relationships through which metabolites and signals move. Grandel et al. [73] summarize this problem as control in composition, time, and space, a framing that captures the main engineering constraints of community-based biosensing.

Compositional control determines whether the designed biosensor remains the same device over time. If a degrader strain disappears, indirect sensing fails. If a fast-growing reporter strain dominates, output may increase for ecological reasons rather than because the analyte is present. If a communication strain changes abundance, activation thresholds shift. Grandel et al. [74] addressed this problem by engineering long-term homeostasis in a two-member microbial consortium through auxotrophic cross-feeding. In their system, mutually auxotrophic *E.coli* strains exchanged missing nutrients, and the population ratio converged to a stable state in continuous culture even when the initial inoculation ratios differed by two orders of magnitude. The ratio could also be tuned by supplying the missing metabolites, and a mathematical model predicted the response of the co-culture to nutrient changes. This supports a key recommendation for biosensor design: community composition should be treated as a controllable circuit parameter before field validation, not as an afterthought.

Temporal control is equally important. Transformation must occur before detection when the target is sensed indirectly. Communication signals must reach useful thresholds without saturating the system. Reporter dynamics must match the assay window. The problem becomes more complex in continuous monitoring, where a stable signal may be more valuable than the strongest possible signal. Essington et al. [75] illustrate this principle in an autonomous microbial sensor for TNT detection in natural soil (Figure 2e). By coupling a TNT-responsive riboswitch to a genetic memory circuit and tuning transcriptional parameters, the engineered *Bacillus subtilis* sensor converted chemical exposure into a persistent output while limiting leakiness and improving dynamic range. Its performance was evaluated over a 28-day soil assay, during which the sensor produced a 14-fold response to low TNT concentrations and maintained stable activation for more than 21 days. This example shows that temporal performance should be evaluated in terms of response durability, circuit burden and longitudinal signal stability, rather than peak reporter intensity alone [76].

Spatial control determines the effective range of interaction. Gupta et al. [77] developed the MISTiC microfluidic platform to control the distance between microbial populations and showed that spatial separation can shape information transmission, oscillator robustness, and auxotroph community stability (Figure 2f). For SynCom biosensors, this means that device architecture is not simply a container. Hydrogel encapsulation, microfluidic channels, membranes, biofilms, patterned agarose, and compartmentalized droplets can alter diffusion, competition, and signal fidelity. The spatial format should therefore be considered part of the biosensor design, especially for real samples with heterogeneous chemistry or high particulate content.

These control principles lead to a practical design rule. Increasing community complexity is justified only when it improves a specific analytical function: broader target coverage, lower burden, better matrix tolerance, improved multiplexing, or more reliable decoding. Otherwise, added strains increase interaction uncertainty and complicate validation. SynCom biosensors should therefore be understood as engineered communities in which functions are assigned across cells, connected through designed interactions, and constrained by compositional, temporal, and spatial control.

### 4.6. Failure Modes and Diagnostic Checkpoints

The same interactions that enable SynCom biosensors can also create characteristic failure modes. Population collapse occurs when an essential member is lost through growth-rate imbalance, toxicity, nutrient limitation, or incompatible culture requirements. Cheater emergence becomes possible when variants benefit from shared metabolites, detoxification, or public goods without maintaining costly sensing, transformation, or communication functions. Mutation accumulation and genetic instability can silence reporters, weaken regulatory circuits, alter secretion or uptake, or disrupt containment modules. Communication breakdown can occur through intermediate dilution, nonspecific consumption, signal degradation, noncognate activation, saturation, or loss of sender-receiver balance. Ecological drift then integrates these effects over time, as gradual changes in population ratio, spatial arrangement, growth state, or matrix adaptation shift the input–output relationship. These risks should be treated as diagnostic checkpoints rather than incidental observations. Failure-mode analysis should include member-specific abundance tracking, reporter stability, plasmid or mutation monitoring, communication-channel testing, strain-ratio perturbation, serial-passage stability, and validation in blank, spiked, mixed-target, and real-sample matrices. A SynCom biosensor should be considered reliable only when its analytical signal remains attributable after these failure modes have been tested.

## 5. AI-Enabled Design, Decoding, and Control of Syncom Biosensors

### 5.1. The Rationale for AI-Assisted Syncom Biosensing

As SynCom biosensors increase in biological and operational complexity, AI is becoming a practical supporting tool rather than a stand-alone solution for guiding their design, optimization, and interpretation. Unlike single-strain WCBs, SynCom biosensors require coordinated decisions across multiple levels, including member selection, functional distribution among strains, intercellular communication or metabolic exchange, community stability, and conversion of heterogeneous outputs into interpretable signals. As the number of strains, genetic circuits, target mixtures, culture conditions, and device formats increases, empirical optimization alone becomes inefficient. AI-assisted approaches can help by prioritizing candidate strains, predicting incompatible interactions, identifying plausible community architectures, extracting informative signal features, and improving the efficiency of the design–build–test–learn cycle [29,78].

The value of AI in this context should be understood as practical rather than rhetorical. AI does not replace experimental validation or ecological reasoning, particularly because current SynCom biosensor datasets remain limited, unevenly annotated, and often specific to particular hosts, targets, or assay formats. Its more realistic role is to make the development process more selective, data-informed, and reproducible. The maturity of these applications is uneven. AI-assisted design prioritization and biosignal decoding are closer to near-term use, whereas closed-loop community control remains largely a prospective strategy for contained systems. This role is especially relevant during candidate discovery. Many useful sensing, transformation, or stress-tolerance functions are found in microorganisms that remain difficult to isolate, cultivate, or characterize by conventional methods. Recent advances in AI-assisted microbial isolation and cultivation indicate a broader transition from experience-driven screening toward data-driven resource discovery across genomic, cellular, and community scales [79]. For SynCom biosensors, such approaches may expand the accessible pool of chassis organisms and functional partners, particularly for applications in complex matrices such as soil, wastewater, food residues, and gut-like environments, where tolerance, metabolic compatibility, and interaction capacity are often as important as the sensing module itself.

### 5.2. Model-Guided Assembly and Interaction Prediction

**Figure 3:**
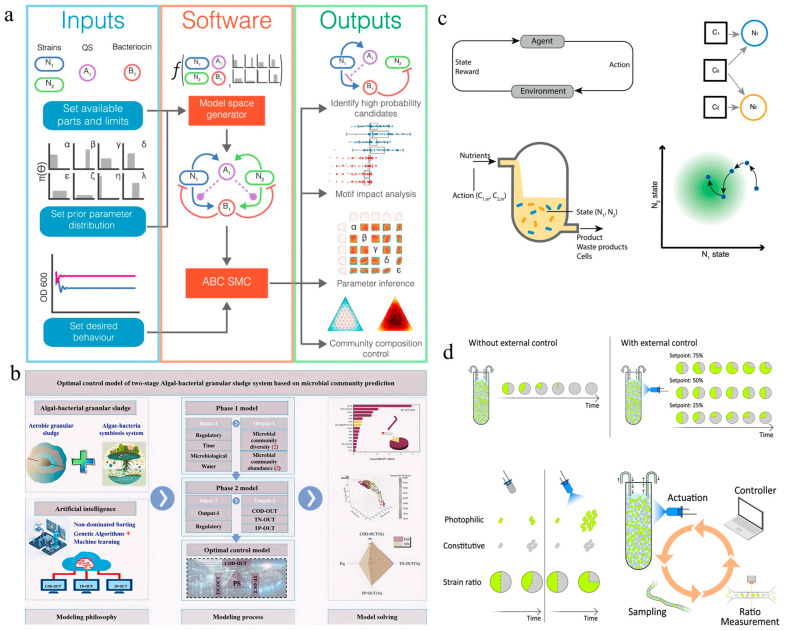
AI-assisted workflows for SynCom biosensor design, decoding, and control. (**a**) AutoCD illustrates computational exploration of synthetic community architectures before experimental construction [30]. (**b**) AI modeling of algae-bacteria granular sludge links community structure, operating conditions, and environmental performance, providing a precedent for data-guided optimization [31]. (**c**) An integrated deep reinforcement learning framework for the in silico control of competitive auxotrophic microbial co-cultures in a continuous bioreactor [34]. (**d**) Schematic of an automated closed-loop optogenetic platform for contained compositional control of bacterial co-cultures [35].

Model-guided assembly is one of the most developed AI-relevant directions in SynCom biosensor design, although it remains closer to design support than to autonomous biosensor construction. Automated and computational approaches can represent nutrient competition, cross-feeding, quorum sensing, inhibition, growth burden, and environmental constraints before strains are built or combined. Karkaria et al. [30] demonstrated this principle through AutoCD (Figure 3a), which generated candidate two- and three-strain communities and used approximate Bayesian computation with sequential Monte Carlo sampling to select designs likely to reach stable steady states. Although AutoCD was not developed specifically as a biosensor platform, it is directly relevant because a biosensor community cannot produce reliable data if its population structure is unstable. Genome-scale metabolic models and related community models provide another route to rational assembly. San León & Nogales et al. [61] reviewed bottom-up, top-down, and hybrid model-based design tools for SynComs, emphasizing that rational design benefits from combining mechanistic models with community-level data. Jing et al. [80] similarly argue that functional SynCom design should integrate high-throughput phenotyping with genomic and metabolic inference. For biosensors, this suggests a workflow in which candidate members are not selected only by taxonomy or availability, but by predicted functional contribution, metabolic compatibility, and ability to generate or preserve interpretable signals. A useful environmental case study comes from Liu et al. [31], who built a two-stage AI framework for an algae-bacteria granular sludge system (Figure 3b). The first stage predicted microbial community succession under different operational conditions, and the second stage connected microbial community structure with pollutant removal performance. The study compared multiple machine-learning algorithms, reported R^2^ values above 0.94 for both stages, and used a non-dominated sorting genetic algorithm to improve carbon, nitrogen, and phosphorus removal, with optimized removal rates exceeding 90%. Although the system was developed for wastewater treatment rather than biosensing, it shows how community composition, process variables, and environmental performance can be linked in a predictive optimization framework. Such examples are best interpreted as transferable design precedents, not as evidence that AI-guided SynCom biosensors have reached field-ready maturity.

### 5.3. Biosignal Decoding and Dataset Design

AI also becomes important after a SynCom biosensor is constructed, especially for interpreting multidimensional outputs that are difficult to separate by simple thresholding. Community biosensors may produce multidimensional signals: multiple fluorescent channels, luminescence kinetics, gas ratios, electrochemical traces, growth curves, spatial fluorescence maps, or temporal response profiles. These outputs often overlap under real sample conditions. Pattern-recognition models can improve classification or concentration estimation, as shown by bacterial bioreporter panels and bacterial sensor arrays in which multistrain luminescence response patterns were decoded by classification algorithms or machine-learning models [32]. Similar logic has been extended to optical and fluorescence sensing systems, where machine learning enables multiplexed signal separation and quantitative interpretation from compact or field-oriented platforms [33,81].

However, such models are useful only if the training data represent the sources of variation expected during deployment. This requirement is stricter for SynCom biosensors than for many single-strain biosensors. A change in signal may reflect analyte concentration, but it may also reflect population drift, nutrient limitation, matrix toxicity, or altered communication thresholds. Machine-learning studies on synthetic communities show that strain identity and community composition can dominate functional outcomes, supporting the need to treat community state as an explicit predictor rather than as hidden noise [82]. For this reason, dataset design should include blank matrices, single-target and mixed-target exposures, negative chemical analogs, strain-ratio perturbations, growth-phase variation, and independent reference measurements. AI decoding should not be trained only on idealized laboratory media. It should be trained to distinguish analyte-dependent signal from community-state variation.

The same principle applies to multiplexed detection. A SynCom biosensor that uses different strains for different analytes will need models that can recognize channel-specific outputs while detecting crosstalk and population imbalance. Ratiometric outputs, internal controls, lineage markers, and member-specific reporters can help generate training labels; stable fluorescent marker systems for SynComs provide a useful precedent for tracking member abundance and assembly dynamics [83]. If the final output is intended for field deployment, the model should be evaluated by sensitivity, specificity, calibration error, false-positive rate in mixed matrices, and robustness to changes in strain abundance, and transferability across batches, devices, and sample types, not only by apparent accuracy on a balanced test set [84].

### 5.4. Adaptive Control During Operation

The most ambitious role for AI is adaptive control, but it is also the least mature for SynCom biosensor deployment. In principle, a SynCom biosensor could adjust medium composition, inducer level, light input, flow rate, incubation time, or sampling frequency to maintain community state and preserve output quality. Treloar et al. [34] used deep reinforcement learning in silico to control microbial co-cultures in bioreactors (Figure 3c), while Gutiérrez Mena et al. [35] demonstrated dynamic cybergenetic control of bacterial co-culture composition through real-time measurement and optogenetic feedback (Figure 3d). These studies are not mature SynCom biosensor implementations, but they provide concrete evidence that community composition can be treated as a controllable variable under modeled or highly instrumented conditions. Adaptive control should be introduced cautiously. It is most realistic in contained devices, microfluidic formats, bioreactors, wearable cartridges, or benchtop monitoring systems where sensors and actuators can be integrated. It is less straightforward in open soils, foods, or environmental waters where actuation is limited and biosafety constraints are stronger. A clearer schematic distinction is therefore needed. Off-line AI can guide design, on-device AI can decode signals, and closed-loop AI may eventually control community state in contained systems with integrated sensing and actuation. Separating these levels avoids overclaiming and keeps the technological progression credible.

## 6. Conclusions and Outlook

The transition from WCBs to SynCom biosensors represents an architectural shift in biosensing, and the further development of AI-assisted SynCom biosensors adds a computational layer to this shift. WCBs demonstrated that living cells can detect bioavailable analytes and convert them into measurable outputs, but their performance becomes constrained when recognition, processing, reporting, stress tolerance, and containment must all be placed in one chassis. Natural microbial consortia show how biological systems distribute functions, coordinate activities, and buffer environmental variation, but they are too context-dependent to serve directly as standardized analytical devices. SynCom biosensors attempt to bridge this gap by assigning recognition, transformation, computation, reporting, and containment to defined community members. AI-assisted workflows may further support candidate selection, interaction prediction, multidimensional signal decoding, and adaptive operation. However, community complexity and computational complexity should be introduced only when they solve a defined analytical problem and preserve an interpretable relationship between input and output.

Three unresolved engineering bottlenecks are likely to determine the pace of the field. The first is community stability and control, because SynCom biosensors must maintain member abundance, functional assignment, and interaction strength across the assay window. The second is analyte-specific signal attribution, because metabolic relay, regulatory communication, and distributed computation can introduce transfer loss, crosstalk, population-dependent thresholds, and matrix-sensitive outputs. The third is deployable validation, because future studies must report member identity, communication channels, spatial format, containment strategy, genetic stability, storage stability, operating procedure, sample pretreatment, matrix tolerance, population stability, quality-control criteria, dataset structure, and model-transfer performance alongside conventional metrics such as sensitivity and detection limit. AI-assisted design and decoding may help address these bottlenecks, but current datasets remain limited, matrix-specific, and difficult to transfer across organisms, devices, and deployment environments.

Scalability and manufacturability are equally important for field deployment. A SynCom biosensor is more difficult to manufacture than a single-strain WCB because the final product must preserve cell viability, member ratio, interaction strength, genetic stability, readout calibration, and, when AI is used, consistency between the biological device and the trained model. Scale-up can alter growth competition, metabolite exchange, diffusion distance, reporter kinetics, and signal distributions, so communities optimized in microplates may not behave identically in cartridges, hydrogels, films, beads, or microfluidic devices. Field-ready systems also require shelf-stable formulation, batch-to-batch reproducibility, simple activation, limited cold-chain dependence, low-cost readout, model robustness to device variation, and safe disposal [85]. These requirements favor contained formats such as immobilized cells, hydrogel-confined communities, membrane-separated co-cultures, microfluidic chips, bioelectronic cartridges, or rehydratable starter formats, but these formats may also reduce mass transfer, weaken signal intensity, shift community composition, and change the feature space used for AI decoding.

In the short term, the most realistic applications are not fully autonomous open-environment biosensors, but contained or semi-contained platforms in which community composition, storage, signal transfer, readout, and data interpretation can be controlled. Promising directions include cartridge-based or hydrogel-confined biosensors for environmental and food-sample prescreening, microfluidic or bioelectronic co-culture devices for water and wastewater monitoring, relay-based systems for analytes that require biological transformation before detection, and process-monitoring formats in bioreactors, fermentation systems, or engineered wastewater units. In these settings, AI is most realistic as a tool for design prioritization, signal decoding, quality control, and decision support rather than fully autonomous field control. In this restrained view, AI-assisted SynCom biosensors are not a universal replacement for instrumental analysis or single-cell biosensors. Their promise lies in applications where biological transformation, multiplexed sensing, environmental resilience, distributed computation, and data-assisted interpretation provide a genuine advantage, and where the device format can preserve community function during production, storage, transport, operation, and disposal.

## Figures and Tables

**Table 1:** Comparative overview of biosensing architectures from WCBs to AI-assisted SynCom biosensors.

Category	Architecture and Functional Logic	Main Advantages	Main Limitations	Representative References
WCBs	Single-cell architecture integrating recognition, signal processing, and reporter output.	Simple, programmable, low-cost, and suitable for bioavailable analyte detection with diverse readouts.	Single-chassis burden, crosstalk, limited multiplexing, matrix interference, and reduced stability in complex samples.	[6,10,11,16]
Natural consortia	Ecological architecture based on naturally distributed functions, including division of labor, cross-feeding, communication, spatial organization, and stress buffering.	Ecological templates for distributed sensing, substrate transformation, robustness, and matrix adaptation.	Context dependence, poor standardization, population drift, and weak attribution between input and output.	[12,13,17,18,19,20,21]
SynCom biosensors	Defined community architecture with assigned member roles and engineered or selected interactions; engineered co-cultures represent minimal or transitional SynCom biosensors.	Functional partitioning, reduced cellular burden, metabolic relay, indirect sensing, matrix tolerance, and multiplexing.	Composition control, signal-transfer efficiency, crosstalk, spatial dependence, long-term stability, biosafety, and real-sample validation	[22,23,24,25,26,27,28,29]
AI-assisted SynCom biosensors	Data-guided community architecture integrating AI-assisted design, interaction prediction, biosignal decoding, and feedback control.	Design-space reduction, improved interaction prediction, multidimensional signal interpretation, and adaptive operation.	Limited datasets, weak transferability, insufficient interpretability, matrix-specific models, and need for external validation.	[15,30,31,32,33,34,35,36]

## Data Availability

No new data were created or analyzed in this study.
